# A stretchable triboelectric nanogenerator made of silver-coated glass microspheres for human motion energy harvesting and self-powered sensing applications

**DOI:** 10.3762/bjnano.12.32

**Published:** 2021-05-03

**Authors:** Hui Li, Yaju Zhang, Yonghui Wu, Hui Zhao, Weichao Wang, Xu He, Haiwu Zheng

**Affiliations:** 1 School of Physics and Electronics, Henan University, Kaifeng 475004, China; 2 School of Mechanical and Automotive Engineering, Henan Key Laboratory for Advanced Silicon Carbide Materials, Kaifeng University, Kaifeng, 475004, China; 3 Defense Key Disciplines Lab of Novel Micro-nano Devices and System Technology, Chongqing University, Chongqing, 400044, China

**Keywords:** human motion energy, silver-coated glass microsphere, single-electrode mode, triboelectric nanogenerator, wearable

## Abstract

Wearable triboelectric nanogenerators (TENGs) have recently attracted great interest because they can convert human biomechanical energy into sustainable electricity. However, there is a need for improvement regarding the output performance and the complex fabrication of TENG devices. Here, a triboelectric nanogenerator in single-electrode mode is fabricated by a simple strategy, which involves a sandwich structure of silicone rubber and silver-coated glass microspheres (S-TENG). The S-TENG exhibits a remarkable performance in harvesting human motion energy and as flexible tactile sensor. By optimizing the device parameters and operating conditions, the maximum open-circuit voltage and short-circuit current of the S-TENG can reach up to 370 V and 9.5 μA, respectively. The S-TENG with good stretchability (300%) can be produced in different shapes and placed on various parts of the body to harvest mechanical energy for charging capacitors and powering LED lights or scientific calculators. In addition, the good robustness of the S-TENG satisfies the needs of reliability for flexible tactile sensors in realizing human–machine interfaces. This work expands the potential application of S-TENGs from wearable electronics and smart sensing systems to real-time robotics control and virtual reality/augmented reality interactions.

## Introduction

Traditional batteries cannot provide a durable and reliable power supply for small portable electronic devices, personalized healthcare, and Internet-of-Things (IoT) devices [1–6]. Thanks to the progress in low-power technology, the power consumption of microelectronic devices has dropped to the level of micro- or nanowatts, which makes the use of environmentally friendly energy a good and practical strategy. Multiple sources of energy could be used, such as wind energy [7], solar energy [8], thermal energy [9], electromagnetic energy [10], and mechanical energy [11], among which mechanical energy is created almost everywhere. Mechanical energy has many obvious advantages over other energy forms, such as high energy density, wide distribution, and simple acquisition. Regarding this, it is desirable to develop wearable devices that convert mechanical energy from human body motion into electricity [12].

Triboelectric nanogenerators (TENGs), with a wide range of material choices and simple device structures, capture the energy of human motion in real time [13]. This form of energy conversion can not only provide sustainable power for electronic systems, but also provide reliable solutions for active sensing and human–computer interfaces [14]. A stretchable TENG with double-helix structure was previously designed. It consisted of silver-coated glass microspheres (SCGMs) and silicone rubber as stretchable conductive thread (SCT) and a silicone rubber-coated SCT as the other triboelectric thread [15]. This TENG can convert the biomechanical energy from human joint motions. The elastomer matrix guarantees that the TENG can be applied in stretchable electronic systems. The TENG generates an open-circuit voltage of 3.82 V and a short-circuit current of 65.8 nA. There are two more references focused on stretchable TENGs utilizing SCGMs to harvest biomechanical energy [16–17]. Zhang et al. invented a closed-structure TENG made of stretchable materials for harvesting human motion energy and monitoring [17]. It can produce an open-circuit voltage up to 150 V and an optimal instantaneous power density of 44.6 mW/m^2^. From the abovementioned references [15–16], it can be seen that the output performance remains to be improved despite the complex fabrication procedure of the TENG devices. More recently, Qian et al. proposed a nylon-regulated TENG in contact-separated working mode, whose open-circuit voltage and short-circuit current can reach up to 1.17 kV and 138 µA, respectively [16]. Although this is a tremendous advancement in output performance, the contact-separated working mode with double electrodes makes it difficult to connect the current conducting wires to moving objects when trying to harvest mechanical energy. Besides, the organic–inorganic composites prepared by embedding relatively hard SCGMs into a soft silicone rubber matrix reduce the stretchability of the TENG devices, which is adverse regarding wearables. Therefore, further investigations to enhance the stretchability of TENGs by innovative design are still required.

In this work, we developed a single-electrode mode, stretchable triboelectric nanogenerator (S-TENG) using a simple strategy. The single-electrode mode enables the TENG to scavenge energy from the irregular mechanical motion of a free object, independent of electrode position and shape [18–19]. The S-TENG with sandwich structure consists of silicone rubber and SCGMs. The latter are either used as electrode or triboelectric layer. The S-TENG can charge commercial capacitors and power LED lights and scientific calculators and has three distinct advantages. These advantages are: (a) It can be made into multiple shapes and placed on various parts of the body to harvest mechanical energy, requires only a simple fabrication process, (b) it shows stable output and long working life, which provides sustainable electricity, and (c) due to the unique structural design of the device and the high elasticity of the silicone rubber, the S-TENG can be stretched easily to 300% to realize a conformal assembly in stretchable electronic systems. The distinct advantages of the S-TENG indicate broad application prospects in wearable electronics and smart sensing systems.

## Results and Discussion

**Figure 1:**
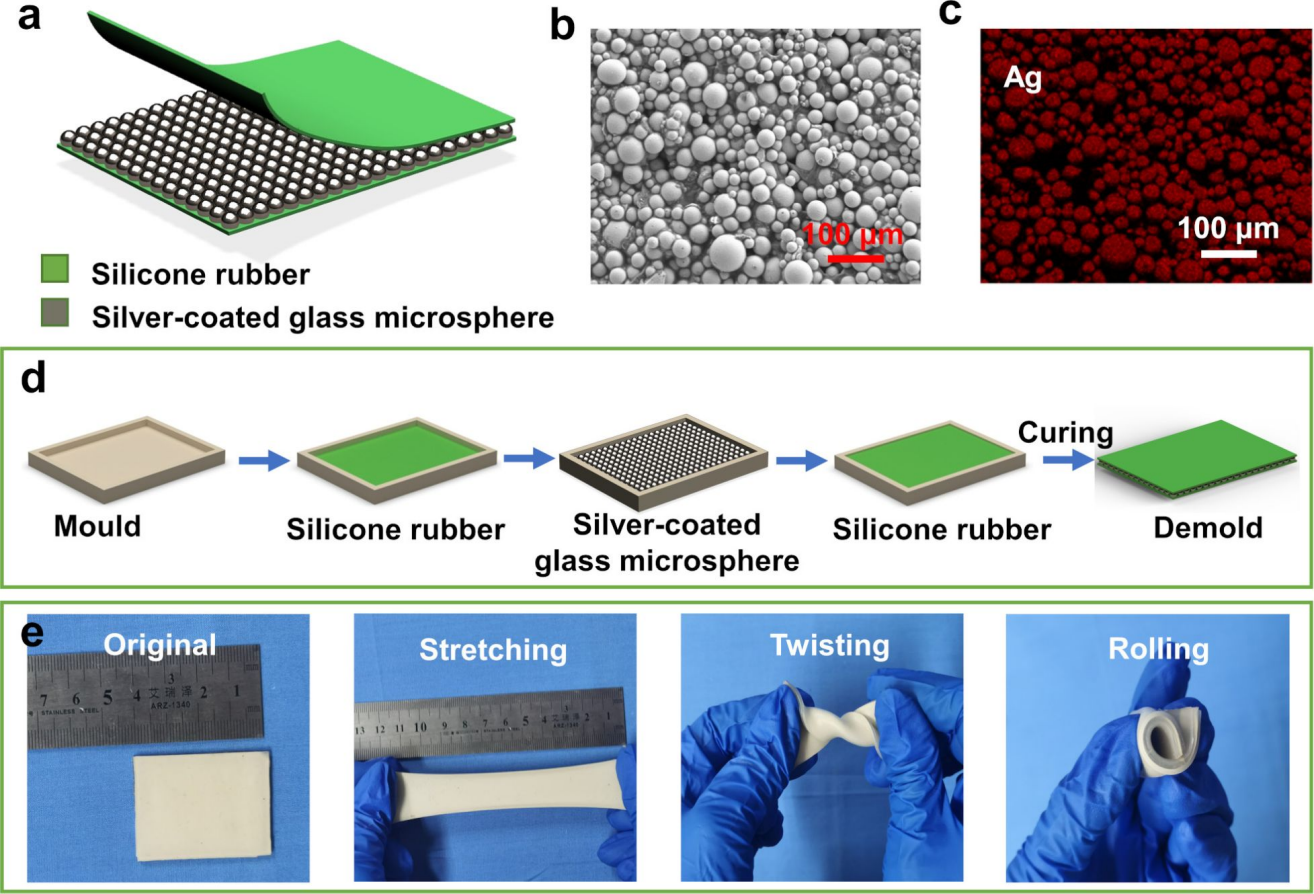
(a) The structural design of the single-electrode mode TENG. (b) SEM and (c) EDS measurements of the SCGM surface. (d) Flow chart for the fabrication process of the S-TENG. (e) Photographs of the S-TENG (original, stretched, twisted and rolled).

Figure 1a is the schematic of the structural design of the S-TENG. The device is composed of three layers, that is, the top layer and the bottom layer of silicone rubber and the middle layer of SCGMs. The scanning electron microscopy (SEM) image (Figure 1b) and the energy dispersive X-ray spectroscopy (EDS) measurement (Figure 1c) show that the SCGMs are evenly dispersed across the silicone rubber. Figure 1d presents the flow chart for the fabrication of the S-TENG. Silicone rubber and SCGMs are filled in a 3D-printed mold and cured. After that, the device is removed from the mold.

The S-TENG can be cut into different shapes for possible applications. The device can be stretched to 300% of the initial length (Video 1, Supporting Information File 1). Also, it can be rolled and twisted easily, as shown in Figure 1e.

**Figure 2:**
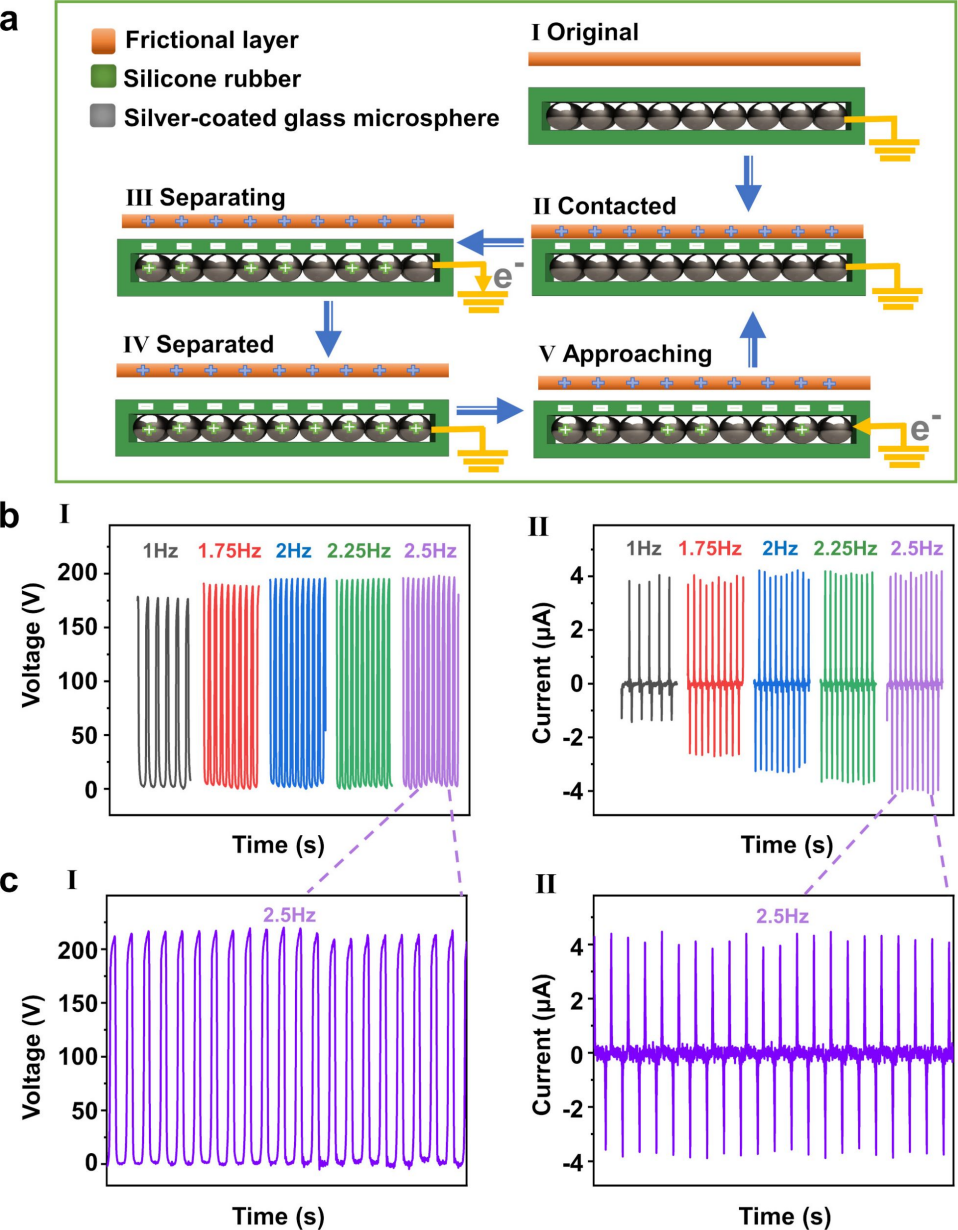
(a) Schematic of the working principle of the S-TENG. (b-I) Open-circuit voltage and (b-II) short-circuit current of the S-TENG at different motion frequencies and an applied force of 90 N. Magnified curves of (c-I) open-circuit voltage and (c-II) short-circuit current when tapping at a motion frequency of 2.5 Hz.

Figure 2a illustrates the electricity generation mechanism of the single-electrode mode S-TENG, which is based on a conjunction of contact triboelectrification and electrostatic induction [2,20–23]. In the initial state (Figure 2a-I), the frictional layer and the S-TENG are in balance without potential difference. When the frictional layer contacts the silicone rubber (Figure 2a-II), the positive charges in the frictional layer are equal to the negative charges in the silicone rubber. Once the surfaces of frictional layer and silicon rubber are separating (Figure 2a-III), the negative charges on the silicone rubber surface drive the electrons of the SCGMs to flow to ground, generating a reverse triboelectric potential. When frictional layer and silicone rubber are entirely separated, the electrostatic equilibrium between the SCGMs and ground is re-established, no output signal can be observed (Figure 2a-IV). When the frictional layer is close to the silicone rubber, the electrons will transfer from ground to the SCGMs and generate a positive potential through the triboelectric effect. Finally, the charge distributions of the two surfaces return to the initial stage.

In order to measure the electrical output of the S-TENG, a piece of 30 × 40 mm^2^ was stuck onto an acrylic plate that was fixed on a linear motor. With the linear motor, displacement and motion frequency of the other triboelectric layer relative to the silicon rubber can be controlled. The open-circuit voltage (*V*_OC_) peaks remain unchanged when the frequency varies from 1 to 2.5 Hz (Figure 2b-I). The short-circuit current (*I*_SC_) increases from 1 to 4.2 μA when the frequency goes from 1 to 2.5 Hz (Figure 2b-II). The peak values of *V*_OC_ and *I*_SC_ go up to nearly 200 V and 4.2 μA, respectively, at 2.5 Hz, as shown in Figure 2c-I and 2c-II. Based on Maxwell’s displacement current, with the increasing number of contact/separation cycles during a unit of time, the charge movement rate between electrode and ground is increasing. Therefore, the *I*_SC_ of the S-TENG can be increased at high frequencies.

**Figure 3:**
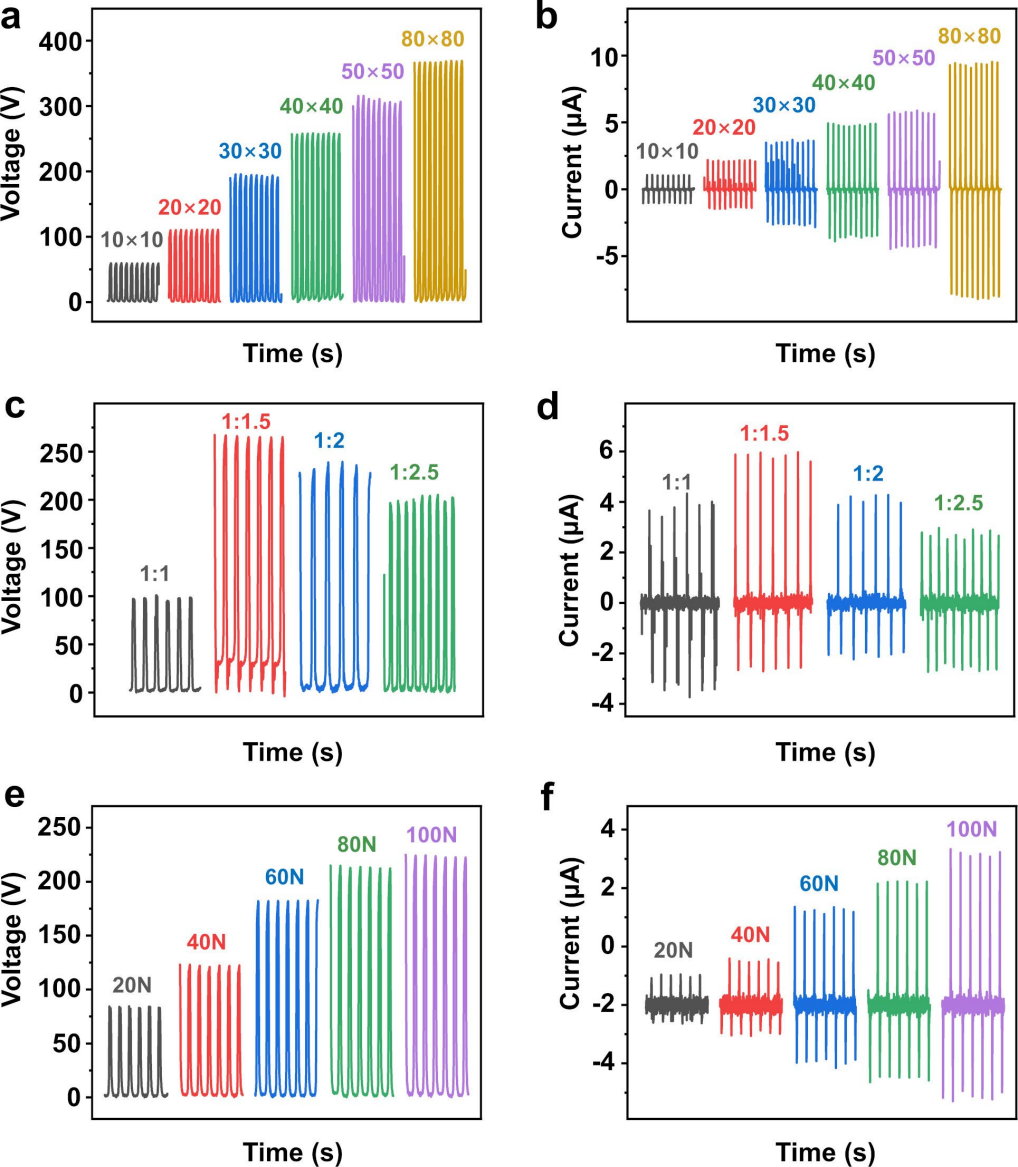
Open-circuit voltage and short-circuit current of the S-TENG (a, b) as functions of the contact area, (c, d) as function of the ratio between silicone rubber and silver-coated glass microspheres, and (e, f) as function of the applied force.

The size plays a crucial role regarding the electrical output of the S-TENG. The output of the S-TENG was studied while changing the size from 10 × 10 mm^2^ to 80 × 80 mm^2^. As shown in Figure 3a and Figure 3b, under a force of 50 N, both *V*_OC_ and *I*_SC_ increase when the contact area increases. When the contact area is 80 × 80 mm^2^, *V*_OC_ and *I*_SC_ reach up to about 370 V and 9.5 μA, respectively. The S-TENG can be used as a large wearable device. With bigger contact area, more charges and, consequently, higher *I*_SC_ values are generated. *V*_OC_ and *I*_SC_ both increase proportionally to the contact area from 10 × 10 mm^2^ to 50 × 50 mm^2^. However, *V*_OC_ and *I*_SC_ of 80 × 80 mm^2^ are not proportionally increased. This may be attributed to the fact that the large device collapses easily in a non-uniform manner. Specifically, the upper and lower surfaces may not contact or separate efficiently, which causes the actual contact area to be smaller than expected [17].

Different devices were prepared by adjusting the ratio between silicone rubber and SCGMs (1:1, 1:1.5, 1:2, 1:2.5). As can be seen from Figure 3c and Figure 3d, when the mass ratio between silicone rubber and SCGMs is 1:1.5, *V*_OC_ and *I*_SC_ reach the largest values of 250 V and 6 μA, respectively, under a force of 150 N. As the content of SCGMs continues to increase, *V*_OC_ and *I*_SC_ gradually decrease. The larger amount of SCGMs causes less air in the same volume. Hence, there is less friction between silicone rubber and SCGMs.

The applied force is another factor that impacts *V*_OC_ and *I*_SC_. As shown in Figure 3e and Figure 3f, when the applied force is increased from 20 to 100 N, the *V*_OC_ values increase from 85 to 225 V and the *I*_SC_ values rise from 1 to 5.3 μA. The reason is that the stronger compressive force leads to an intensification of friction and the generation of more charges. A similar trend is observed for *V*_OC_, as expected.

**Figure 4:**
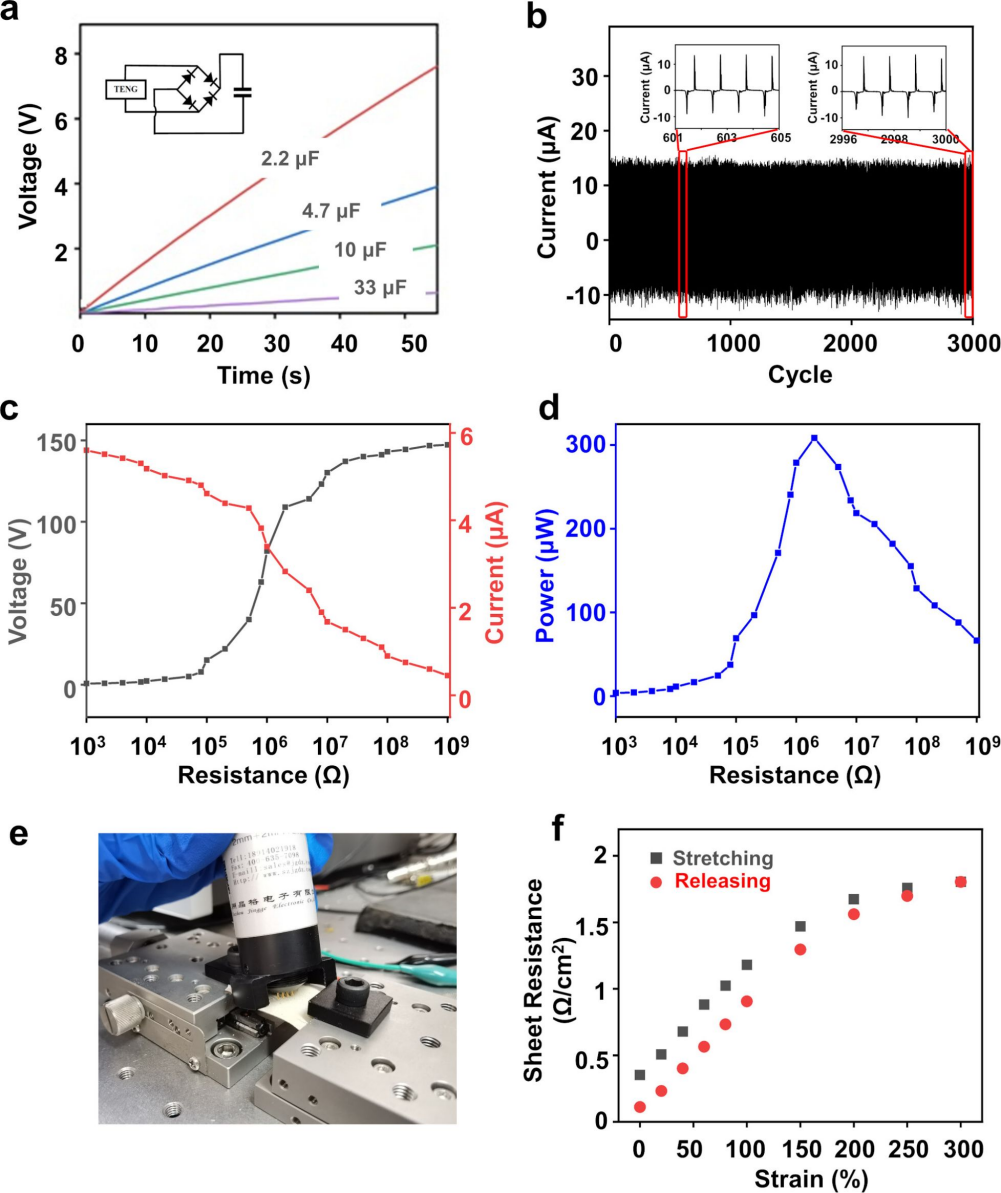
(a) Voltage of different capacitors (2.2, 4.7, 10, and 33 μF) as function of the charging time with the rectified S-TENG output. The inset shows the circuit diagram of the charging system. (b) Cyclic stability of the S-TENG for about 3000 cycles. The inset shows enlarged vies of the middle and the last five cycles. (c) Voltage/current trends and (d) output power of the S-TENG under different load resistances. The maximum power is 308 μW under an external load resistance of 2 MΩ. (e) Photograph of the custom-made resistance test platform. (f) Sheet resistance under tensile strain from 0 to 300%.

Because of the mismatch between AC and DC systems a full-wave rectifier circuit was introduced to the setup. Figure 4a shows the voltage of different capacitors (2.2, 4.7, 10, and 33 μF) as function of the charging time with the rectified S-TENG output. The voltage reaches a saturation value of 14 V for charging a 2.2 μF capacitor.

Reliability is a key parameter considering the practical application of the S-TENG. As depicted in Figure 4b, the *I*_SC_ values of the device do not decline after 3000 cycles for 25 min, demonstrating the long-term stable operation of the S-TENG on the human body. The impedance matching experiment was designed to measure the powering capability of the S-TENG with different resistances. Figure 4c,d shows the relationship between load resistance and *I*_SC_, *V*_OC_, and power of the S-TENG (30 × 40 mm^2^). With the resistance in parallel changing from 1000 Ω to 1 GΩ, *V*_OC_ increases from 0.6 to 147 V and *I*_SC_ decreases from 5.6 to 0.45 μA. The output power (blue line) is calculated as *P* = *U*·*I*. The instantaneous power can achieve a peak value of 308 μW (*V*_OC_ = 109 V and *I*_SC_ = 2.8 μA) with an external load resistance of 2 MΩ. As can be seen from Figure 4e and Figure 4f, the resistance linearly increases from 0.35 to 1.18 Ω/cm^2^ when the tensile strain reaches 300%. In the process, the thickness of the SCGM layer becomes thinner and the resistance of the S-TENG increases linearly. When the S-TENG is further stretched, the slope of the curve falls again because the relative variation of the SCGM layer decreases. After releasing the strain, the resistance is recovered at 0.11 Ω/cm^2^ resulting from a hysteresis in the rearrangement of the SCGMs. Although the curves between sheet resistance and tensile strain have different shapes, the resistance value before and after stretching is of the same order, exhibiting the excellent flexibility and mechanical robustness of the S-TENG.

### Applications of S-TENG

#### Charging performance and monitoring human motion

**Figure 5:**
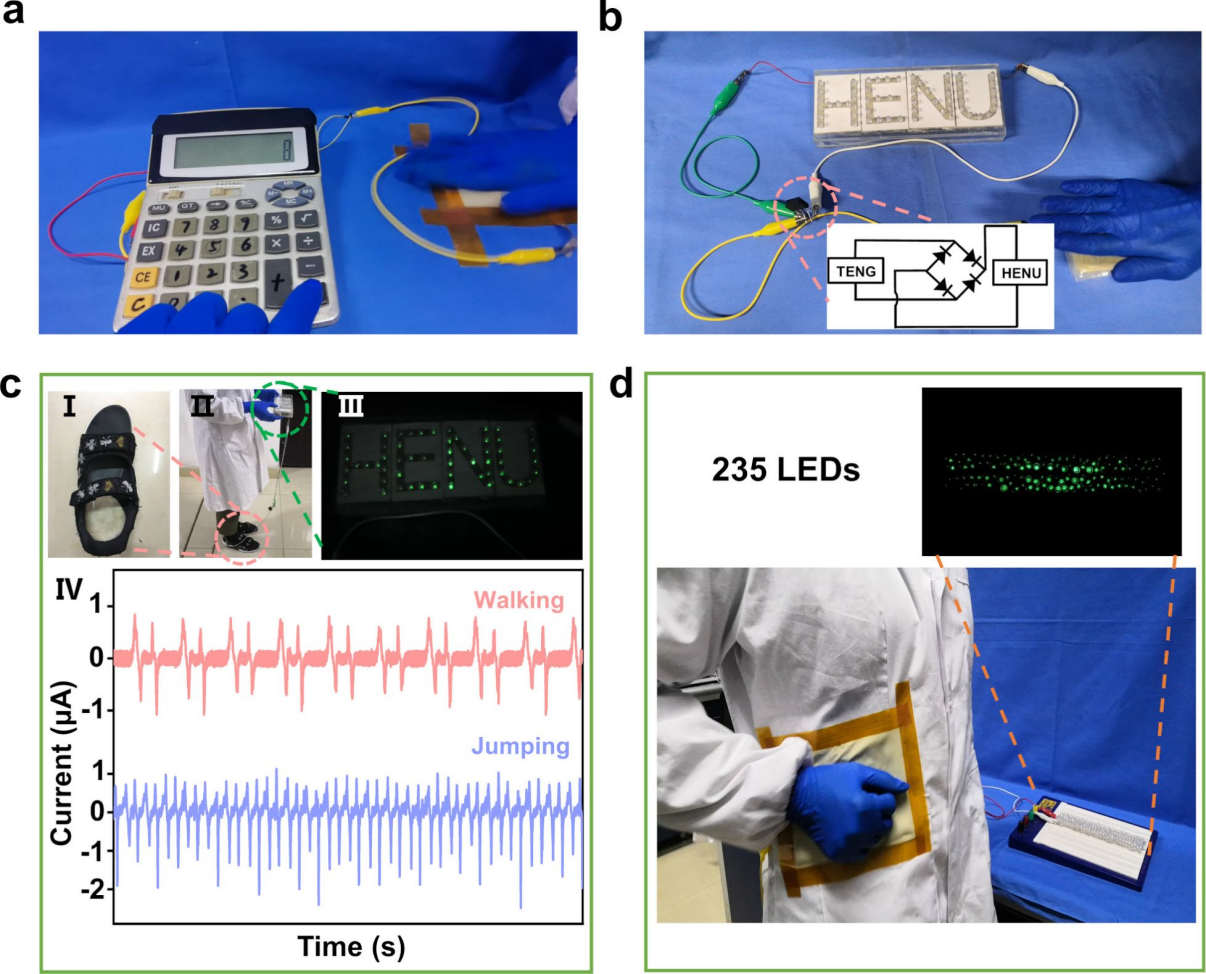
Optical image of the power supplied by padding the S-TENG with the hand for (a) a calculator and (b) LEDs showing “HENU”. The inset shows the circuit diagram of the rectifier. (c) Demonstration of the S-TENG to detect human motion; (I, II) the position of the S-TENG in a shoe, and (III) powered LEDs; (IV) current output of the S-TENG during the two different motions. (d) The S-TENG can be fixed at the waist of a human and can light up 235 LEDs.

The electrical output of S-TENGs has been used to power small electronic components [17]. In Video 2 (Supporting Information File 2) the S-TENG is connected to a linear motor. 235 LEDs connected in series can be lit up after rectification of the output. Because of the video frame rate, some LEDs that light up are not captured. Figure 5a and Video 3 (Supporting Information File 3) show that a scientific calculator is powered on after padding the S-TENG for eight seconds. If the S-TENG device continued to generate electrical output, the calculator would work for a longer time when needed. Figure 5b and Video 4 (Supporting Information File 4) show that LEDs showing the word “HENU” are lit when they are connected to the S-TENG.

The S-TENG can be put into shoes to monitor human movement [24]. An S-TENG device with a diameter of 50 mm and a thickness of 3 mm is suitable to be worn regularly. As shown in Video 5 (Supporting Information File 5), the primary movement of a human being is recorded. The current of the S-TENG for walking and jumping is 1.5 and 2 μA, respectively, as shown in Figure 5c. Two forward electrical signals can be captured every one second during walking. From the graph, the backward-signal *I*_SC_ generated by the three exercise modes is higher than the forward-signal *I*_SC_. The air mixed with SCGMs of the S-TENG is squeezed during the process of stamping continuously. Thus, the S-TENG cannot be recovered to the original state. Different motions produce different signals. The *I*_SC_ generated by jumping is higher than that generated by walking, due to the larger applied force under intense exercise conditions.

The S-TENG device can be potentially applied to harvest energy from different human motions and yield motion statistics. These data can be used for the analysis of physical exertion and exercise intensity by using a micro-processing unit that includes analog-to-digital conversion and wireless transmission. From the number of steps a person takes each day, the number of calories burned through exercise can be estimated. A person can keep trying to lose weight using the device. Also, the S-TENG device can monitor the rehabilitation training of post-op patients who need to avoid excessive physical training that may be harmful to heart or lung. It can also be used to check heartbeat and breathing rate, which are low-intensity movements, for comprehensive health analysis and evaluation [17].

The S-TENG can be placed in different positions of the human body to harvest motion energy with stimulation from another triboelectric layer, such as clothing or the hand. An S-TENG with an area of 100 × 100 mm^2^ was placed on the waist of a person. As shown in Figure 5d and Video 6 (Supporting Information File 6), the S-TENG can light up 235 LEDs by the continuously padding the device in single-electrode contact-separation mode. The S-TENG also can be placed on elbow and knee joints and harvest body motion energy for wearable devices [25].

#### Sensing applications

**Figure 6:**
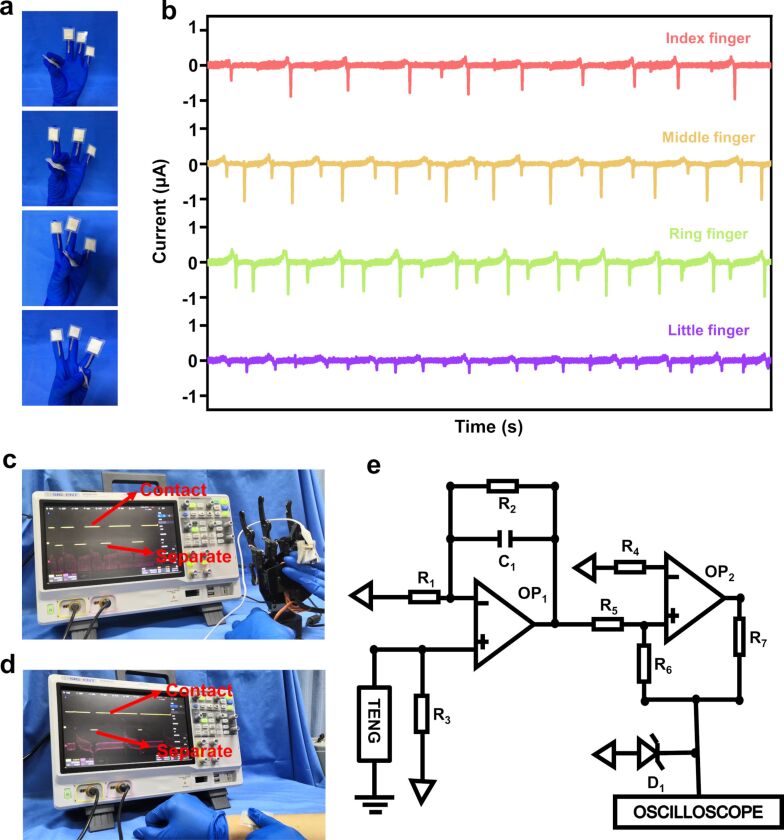
(a) The position of the S-TENGs on the fingers. (b) Current output of the thumb touching the other fingers. (c, d) Potential application of the S-TENG in robotic sensing. (e) The circuit diagram of the signal processing unit.

The S-TENG provides an effective power source for electronic devices. Another potential application for the S-TENG is as flexible tactile sensor that can serve as electronic skin for a more comfortable interactive experience between humans and external objects by sensing all kinds of information, such as size, shape, and texture [26–27]. The flexible tactile sensor can generate electrical signals in response to different mechanical stimuli for the self-supply with energy. An S-TENG with an area of 20 × 20 mm^2^ was placed on each of five fingertips, as exhibited in Figure 6a. When the thumb touches index finger, middle finger, ring finger and small finger in turn, the resulting *I*_SC_ is 1, 1.2, 0.8, and 0.3 μA, respectively, as depicted in Figure 6b.

A single-electrode S-TENG was installed on the finger of a robotic hand [26], as shown in Figure 6c,d and Video 7 and Video 8 (Supporting Information File 7, Supporting Information File 8). An electrical signal is generated when the robot touches an object. When the output signal passes through the signal-processing circuit, the digital waveform can be obtained. The current level (high or low) indicates whether finger and object are in contact. Further data processing can realize basic contact perception. Figure 6e shows the circuit diagram of the signal-processing unit.

## Experimental

In this work, a simple method for fabricating a TENG is proposed. First, a layer of cured silicone rubber film (Smoth-on, Ecoflex 00-20) was prepared. Gel A and gel B were mixed together with a volume ratio of 1:1 in a container for 5 min. Then the mixture was poured into a mold with rectangular groove (30 × 40 mm^2^) manufactured by a 3D printer, and a vacuuming operation was performed for 5 min to eliminate any entrapped air. After curing for 30 min at 50 °C, the rectangular silicone sheet was peeled off from the mold slowly. Second, SCGMs (diameter of 30 μm, Shenzhen Changxinda Shielding Materials Co. LTD) with good electrical conductivity were spread evenly over the surface. Finally, different films were prepared by adjusting the ratio between silicone and rubber/SCGMs (1:1, 1:1.5, 1:2, 1:2.5).

### Characterization and measurements

The values of *V*_OC_ and *I*_SC_ were measured by using an electrometer (Keithley 6514) and the data acquisition device. The force was applied by a linear motor (Linmot E1100) and a force detector. The TENG was attached onto the fixed end of a linear motor. Sensing measurements used LabVIEW programs to record information. The sheet resistance of the flexible SCGM/silicone rubber electrode was measured using the M-3 Mini four-probe tester. SEM (JSM-7001F) was used for morphology characterization of the surface of the SCGMs.

## Conclusion

In summary, an easily manufactured, inexpensive and stretchable single-electrode mode S-TENG was designed and fabricated, of which the electrode was made of a conductive fabric. The top and bottom layers of the sandwich structure are silicone rubber and the middle layer are SCGMs both as conducting layer and frictional layer. The peak values of *V*_OC_ and *I*_SC_ of the S-TENG are nearly 200 V and 4.2 μA at a frequency of 2.5 Hz. Moreover, the device has good long-term stability with almost no degradation of the electrical output after 3000 cycles. In addition, the S-TENG can light up 235 LEDs and power a commercial calculator by padding the S-TENG with the hand. The S-TENG device can be made into various shapes and sizes, and can be freely placed on different parts of body to harvest human motion energy. It has been proven that the device is suitable for wearable energy harvesting. A large-scale device could be used to power portable electronic devices.

## Supporting Information

File 1 Video S1: Stretchability of the S-TENG.

File 2 Video S2: Power LEDs.

File 3 Video S3: Power a calculator.

File 4 Video S4: Light up letters of “HENU”.

File 5 Video S5: Placement in a shoe.

File 6 Video S6: Attachment to the waist.

File 7 Video S7: Robotic sensing.

File 8 Video S8: Human–computer interaction.
